# Supplementary material for: Mapping of the fibrinogen-binding site on the staphylocoagulase C-terminal repeat region
Source: J Biol Chem. 2021 Dec 13;298(1):101493. doi: 10.1016/j.jbc.2021.101493 (PMC8761706; doi:10.1016/j.jbc.2021.101493)
Supplement: Supporting Information [file mmc1.docx]

**Supporting information**

**Figure S1**: Alignment of full-length SC C-terminal repeats showing conserved residues in the minimal peptide region


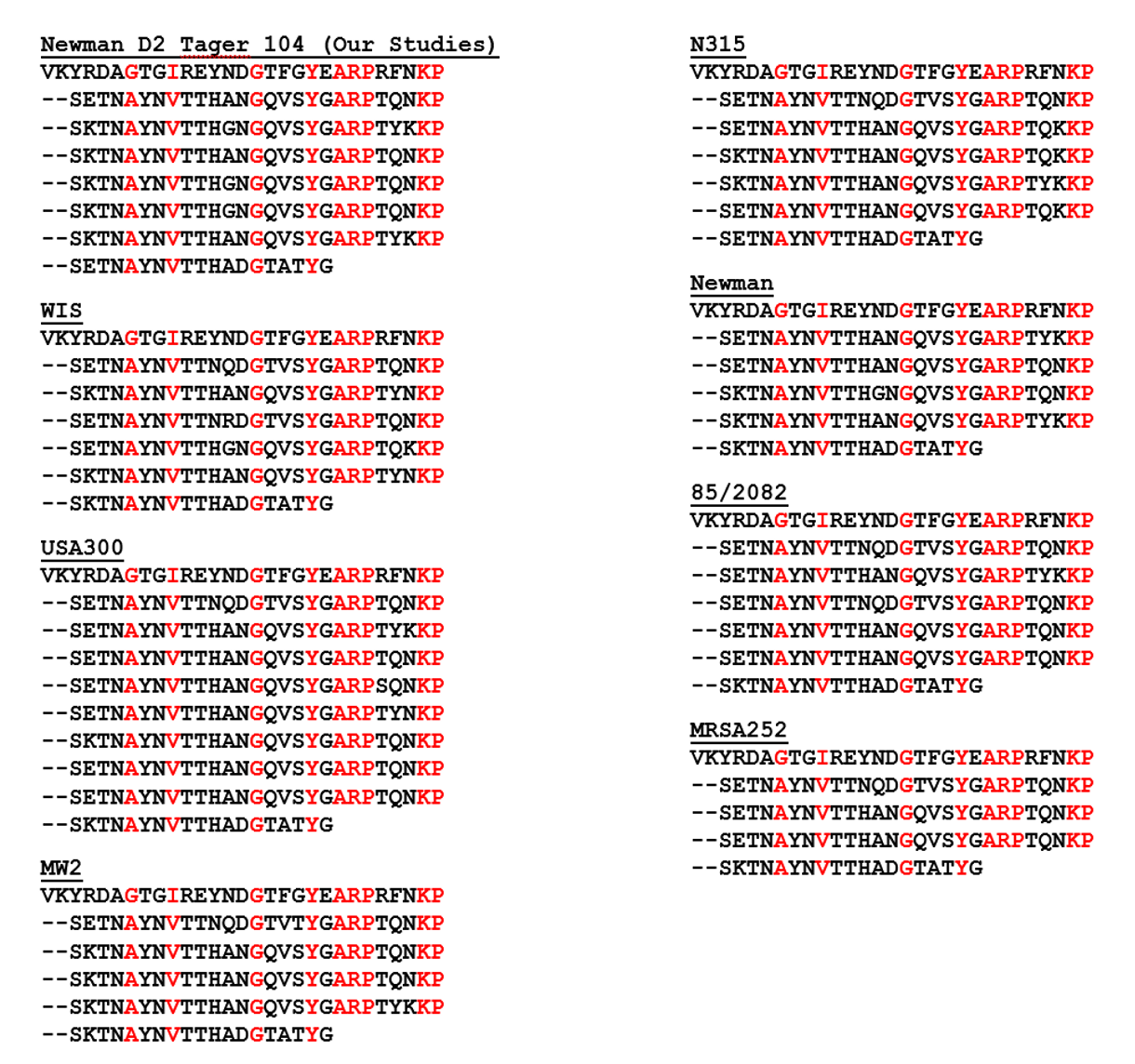


**Figure S2: Native PAGE of Frag D binding to [5F]PR-R1, and lack of binding to [5F]R1 and [5F]PR*. A, B,*** Fluorescence and Coomassie stain of [5F]PR-R1(12.2 µM) incubated with 0.5-, 1.0- and 2.0-fold molar excess Frag D (lanes 2-4) at 25 ^o^C for 30 min. [5F]PR-R1 alone (lane 1) did not enter the gel, but mixtures with Frag D show residual uncomplexed [5F]PR-R1 in lanes 2 and 3 (B, lower bands), and excess uncomplexed Frag D in lane 4 (B, lower band). Lanes 5-8 show the lack of interaction of [5F]R1 (50 µM) with Frag D at 0.5-, 1.0- and 2.0-fold molar excess Frag D, as indicated by the unchanged positions of [5F]R1 alone and in mixtures with Frag D. [5F]R1 alone (lane 5) was detected by fluorescence but stained poorly by Coomassie. Line 9 is a Frag D control. ***C, D,*** Fluorescence and Coomassie stain of [5F]PR alone (lane 1, 45.0 µM) and in mixtures with 0.7-, 1.3- and 2.0-fold molar excess Frag D (lanes 2-4) after 30 min, 25 °C, showing unchanged positions of [5F]PR. Lane 5 is a Frag D control. [5F]PR alone was detected by fluorescence but stained poorly by Coomassie. Samples were run on native 6% Tris-Glycine PAGE at 4 ^o^C.


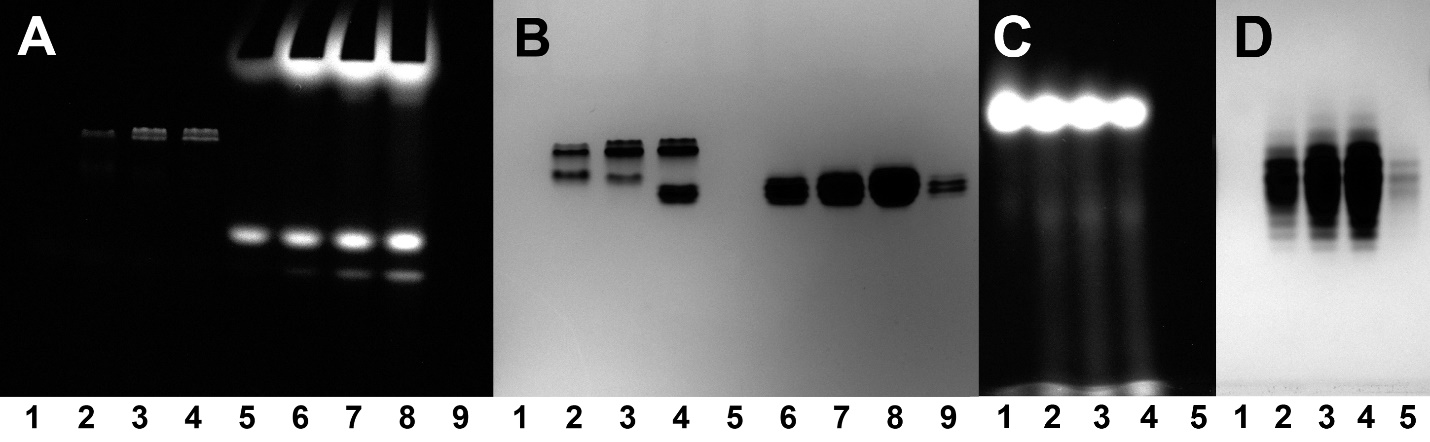


**Figure S3: Binding of Frag D to the SC(1-660)·[5F]ProT complex: *A***, fluorescence and ***B***, Coomassie stain. Incubation of [5F]ProT (2.5 μM) with SC(1-660) (3.7 μM) for 30 minutes, and formation of the SC(1-660)·[5F]ProT binary complex (lane 3). The complex was reacted with Frag D (lanes 4-9, respectively 7.5, 11.2, 15.0, 18.7, 26.2 and 29.9 µM) at 25 ^o^C for 30 minutes to form higher order complexes. Lanes 1, 2 and 10 are [5F]ProT, SC(1-660), and Frag D controls. Proteins were run on a 6 % Tris-Glycine native gel at 4 ^o^C.





**Figure S4: Binding of Frag D to [5F]PR-(R1⭢R7): *A***, fluorescence and ***B***, Coomassie stain of incubations of [5F]PR-(R1⭢R7) (4.3 μM) with Frag D (lanes 2-8, respectively 10.0, 15.0, 20.0, 25.0, 30.0, 35.0 and 40.0 µM) at 25 ^o^C for 30 minutes. Lanes 1, 9 and 10 are respectively [5F]PR-(R1⭢R7) (eluted with dye front and not visible), Frag D control and SC(1-660) as external control. Proteins were run on a 6 % Tris-Glycine native gel at 4 ^o^C.


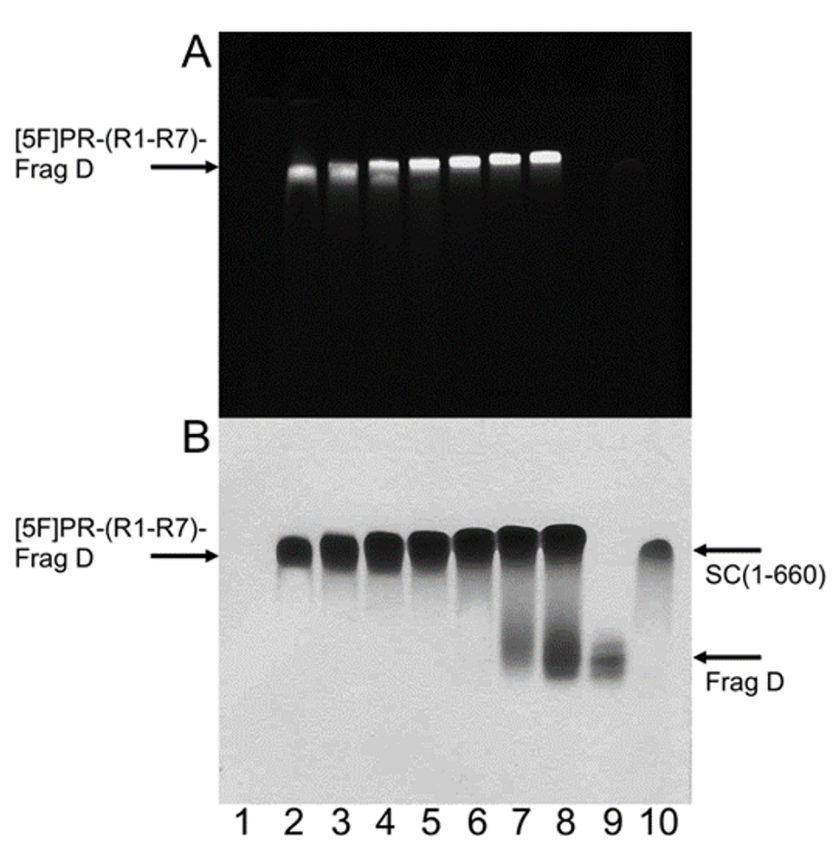


**Figure S5: Binding of Frag D to [5F]PR-R1R6R7 and [5F]PR-R1R2R3: *A***, fluorescence and ***B***, Coomassie stain of incubations of [5F]PR-R1R6R7 (5.6 μM) with Frag D (lanes 2-8, respectively 5.0, 10.0, 20.0, 30.0, 40.0, 50.0 and 60.0 µM) at 25 ^o^C for 30 minutes. ***C***, fluorescence and ***D***, Coomassie stain of incubations of [5F]PR-R1R3R3 (8.1 μM) with Frag D (lanes 2-8, concentrations as above) at 25 ^o^C for 30 minutes. Lanes 1, 9 and 10 in both gels are respectively labeled peptide (eluted with dye front and not visible), Frag D control, and SC(1-660) as external control. Proteins were run on a 6 % Tris-Glycine native gel at 4 ^o^C.





**Figure S6 : PR-R7 residue assignment.** The NMRViewJ Assignment Panel shows assignment status of amide proton and nitrogen, carbonyl carbon, C_α_, C_β_, H_α_, and H_β_. Green squares indicate assigned, black un-assigned atoms. Prolines and glycines serve as checkpoints in the carbon detect methods and are indicated in red or purple, respectively.

**
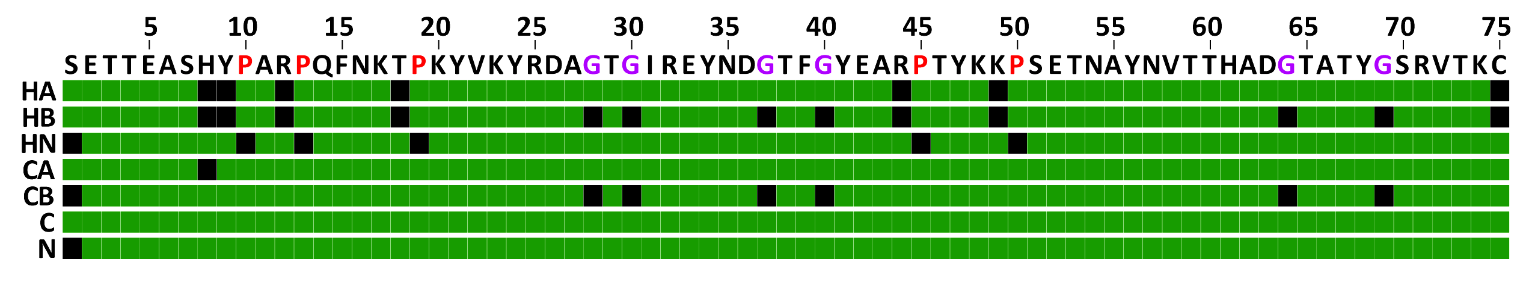
**

**Figure S7: Secondary structure prediction results from Talos+.** The upper panel shows predicted order parameter S2 for each residue and absence of predicted secondary structure for PR-R7. The lower panel shows the classification of each residue: *blue* = dynamic, no prediction; *green* = unambiguous prediction; *yellow* = ambiguous, no prediction.

**
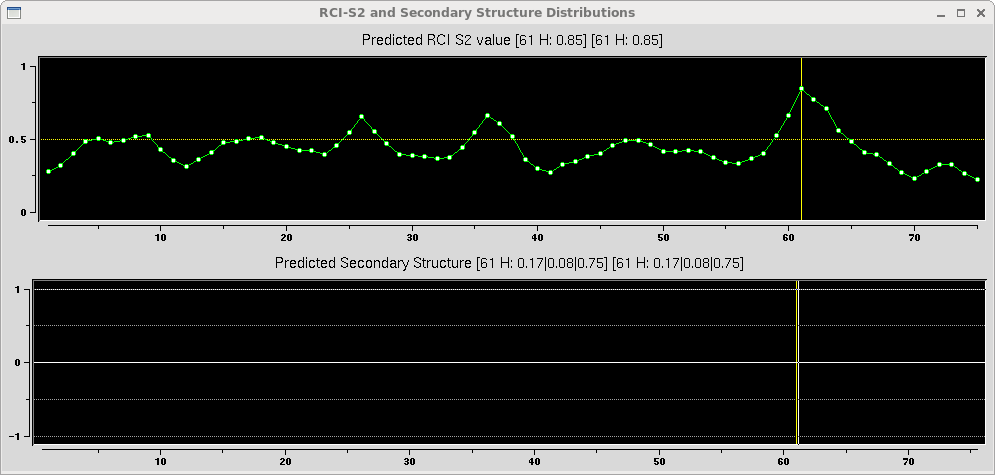
**

**
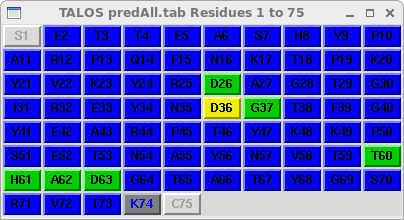
**

**Figure S8**: **Fluorescence anisotropy titrations of repeat constructs binding to Frag D, results with replicate preparations of [5F]PR-(R1⭢R7), PR-R1R2R3 and Frag D: *A***, observed anisotropy (*r_obs_*) of 21 (○) and 155 (●) nM [5F]PR-(R1⭢R7) as a function of total Frag D; ***B***, *r_obs_* of 16 (○) and 120 (●) nM [5F]PR-(R1⭢R7) as a function of total Frag D; ***C***, simultaneous fit of ***A*** with competitor PR-R1R2R3 titrated into mixtures of 21 nM [5F]PR-(R1⭢R7) with 107 (**∆**) and 1012 (**▲**) nM Frag D; ***D***, simultaneous fit of ***B*** with competitor PR-R1R2R3 titrated into mixtures of 16 nM [5F]PR-(R1-R7) with 104 (**∆**) and 1040 (**▲**) nM Frag D. Titrations and data analyses were performed as described under “Experimental Procedures.” *Solid black lines* represent the quadratic (***A***,***B***) and cubic binding fits (***C***,***D***). Binding parameters are given in Table 2.





**Figure S9: Circular Dichroism Spectra of Different SC Proteins and Frag D.** CD spectra of PR-R7 (*black*), PR-(R1⭢R7) (*red*), SC(1-325) (*brown*), SC(1-660) (*blue*), and fragment D (*green*) were measured using a Jasco J-810 spectrometer at 25 ^o^C.

**
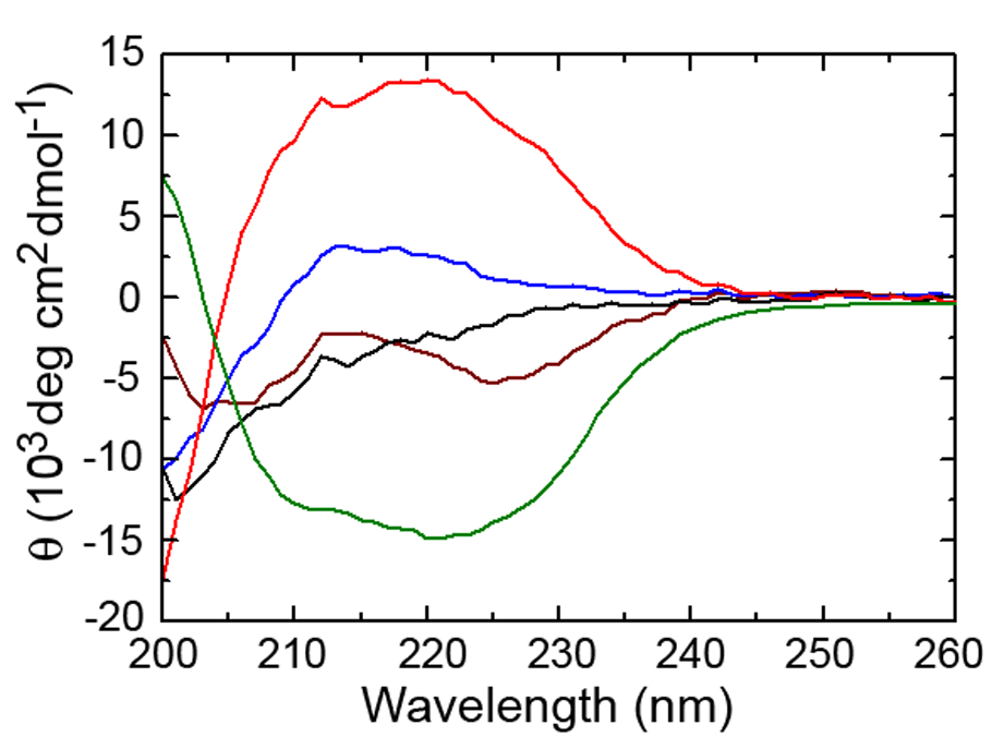
**

**Figure S10: Proposed interaction model of Frag D with the inter-repeat junctions:** A maximum of 7 Frag D molecules may associate with the PR-(R1⭢R7) domain, as suggested by our native PAGE and equilibrium binding titrations. Secondary structure prediction and UV-CD suggest a coil or disordered structure for PR-(R1⭢R7), perhaps allowing larger conformational freedom for multiple Frag D binding. The much larger fibrin(ogen) molecules might associate with these junctions in staggered arrays and favor transition of the disordered SC repeat domain into more organized complex structures such as seen in fibrin protofibril association. Binding of fibrinogen in solution may result in lower stoichiometries than those observed for Frag D binding. In the absence of X-ray or cryo-EM data, structural organization of these higher order complexes remains hypothetical.


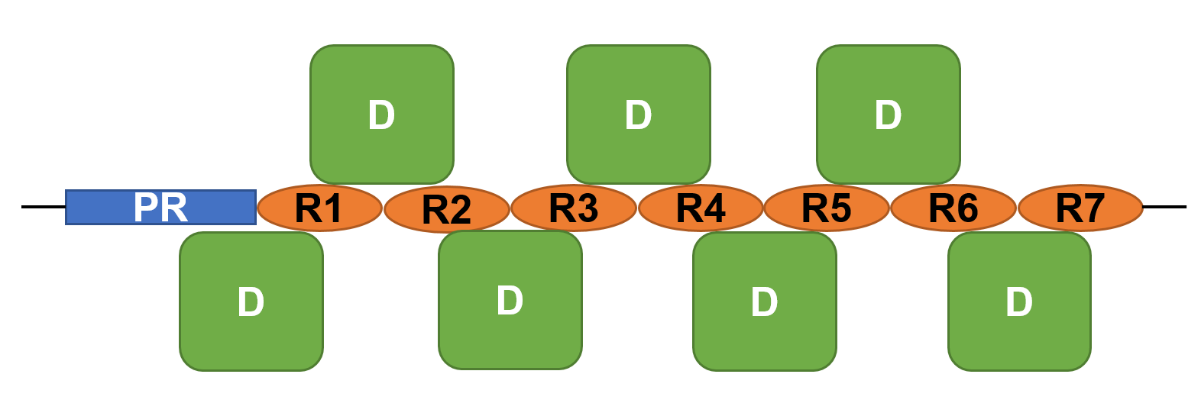


**Supporting Information S11: Quadratic and cubic equations for the analysis of equilibrium binding data**

**A.** Quadratic binding curve, fluorescence intensity data (*F*) from *F*_0_ (initial fluorescence intensity) to *F*_M_ (maximum fluorescence intensity)

Independent variables: L_0_ (ligand), P_0_ (labeled receptor or probe)

Dependent variable: *F*

Parameters: *F*_M_ , *F*_0_ , *K*_D_ , N (stoichiometric factor for L binding to P)

F1 = N*P_0_ + *K*_D_ + L_0_

F2 = F1^2^ - 4*N*P_0_*L_0_

A = F1 - $\surd F2$

*F* = *F*_0_ + A*[(*F*_M_ - *F*_0_)/(2*N*P_0_)]

**B.** Modified quadratic binding curve, with observed anisotropy data (*r*_obs_), ranging from *r*_0_ (initial anisotropy) to *r*_M_ (maximum anisotropy) and corrected for maximum relative fluorescence intensity change F = (*F*_M_/*F*_0_). *F*_0_ is initial fluorescence, and *F*_M_ is maximum fluorescence

Independent variables: L_0_ (ligand), P_0_ (labeled receptor or probe)

Dependent variable: *r*_obs_

Parameters: *r*_M_ , *r*_0_ , F , *K*_D_ , N

F1 = N*P_0_ + *K*_D_ + L_0_

F2 = F1^2^ - 4*N*P_0_*L_0_

A = (F1 - $\surd F2$)/(2*N*P_0_)

*r*_obs_ = [*r*_0_ *(1-A) + (F* *r*_M_ *A)]/[(1-A)+A*F]

0<A<1

**C.** Cubic equation for simultaneous analysis of direct binding (L to P), and competitive binding (L to P and C, competitor), anisotropy data corrected for fluorescence intensity change

Independent variables: L_0_ (ligand), P_0_ (labeled receptor or probe), C_0_ (competitor)

Dependent variable: *r*_obs_

Parameters: N, *K*_0_ (*K*_D_ for L binding to P), M (stoichiometric factor for L binding to C), *K*_C_ (*K*_D_ for L binding to C), *r*_0_ , *r*_M_ , F (= *F*_M_/*F*_0_)

D1 = N*P_0_*(*K*_C_-*K*_0_)/*K*_0_

D2 = L_0_*(*K*_0_-*K*_C_)/K_0_ + N*P_0_*(*K*_0_-2**K*_C_)/*K*_0_ + *K*_0_ – *K*_C_ – M*C_0_

D3 = L_0_*(2**K*_C_-*K*_0_)/*K*_0_ + N*P_0_**K*_C_/*K*_0_ + *K*_C_ + M*C_0_

D4 = L_0_**K*_C_/*K*_0_

D1*A^3^+D2*A^2^+D3*A = D4

*r*_obs_ = [*r*_0_ *(1-A) + (F* *r*_M_ *A)]/[(1-A)+A*F]

0<A<1
